# Correction: Fang et al. In Vivo Rodent Models of Type 2 Diabetes and Their Usefulness for Evaluating Flavonoid Bioactivity. *Nutrients* 2019, *11*, 530

**DOI:** 10.3390/nu15132881

**Published:** 2023-06-26

**Authors:** Jia-You Fang, Chih-Hung Lin, Tse-Hung Huang, Shih-Yi Chuang

**Affiliations:** 1 Pharmaceutics Laboratory, Graduate Institute of Natural Products, Chang Gung University, Kweishan, Taoyuan 33302, Taiwan; fajy@mail.cgu.edu.tw; 2 Chinese Herbal Medicine Research Team, Healthy Aging Research Center, Chang Gung University, Kweishan, Taoyuan 33302, Taiwan; 3 Research Center for Food and Cosmetic Safety and Research Center for Chinese Herbal Medicine, Chang Gung University of Science and Technology, Kweishan, Taoyuan 33302, Taiwan; 4 Department of Anesthesiology, Chang Gung Memorial Hospital, Kweishan, Taoyuan 33302, Taiwan; 5 Center for General Education, Chang Gung University of Science and Technology, Kweishan, Taoyuan 33302, Taiwan; chlin@mail.cgust.edu.tw; 6 Department of Traditional Chinese Medicine, Chang Gung Memorial Hospital, Keelung 20401, Taiwan; huangtsehung@gmail.com; 7 School of Traditional Chinese Medicine, Chang Gung University, Kweishan, Taoyuan 33302, Taiwan; 8 Graduate Institute of Health Industry Technology, Chang Gung University of Science and Technology, Kweishan, Taoyuan 33303, Taiwan; 9 School of Nursing, National Taipei University of Nursing and Health Sciences, Taipei 112, Taiwan

**Missing Citation**

In the original publication [1], refs [2,3] were not cited. The citations have now been inserted in Section 2.4.1, the first paragraph and should read: The rodent has proven to be a reliable model for discovering and validating new treatments for T2D (Table 1) [38]. In Section 2.4.2, the first paragraph should read: Polygenic models of obesity may provide a more accurate model of the human condition [54].

With these corrections, the order of some references have been adjusted accordingly. The authors apologize for any inconvenience caused and state that the scientific conclusions are unaffected. This correction was approved by the Academic Editor. The original publication has also been updated.
